# FMW-YOLO: A Frequency-Enhanced and Multi-Scale Context-Aware Framework for PCB Defect Detection

**DOI:** 10.3390/mi17050531

**Published:** 2026-04-27

**Authors:** Yuguo Li, Shuo Tian, Wenzheng Sun, Longfa Chen, Jian Li, Junkai Hu, Na Meng

**Affiliations:** College of Intelligent Equipment, Shandong University of Science and Technology, Tai’an 271019, China; 202483230068@sdust.edu.cn (Y.L.); 202483230076@sdust.edu.cn (S.T.); 202383230038@sdust.edu.cn (W.S.); 202383230046@sdust.edu.cn (L.C.); 202583230070@sdust.edu.cn (J.L.); 202322020105@sdust.edu.cn (J.H.)

**Keywords:** PCB defect detection, frequency enhancement, Channel-Transposed attention, multi-scale features

## Abstract

A high-precision and efficient surface defect detection for printed circuit board (PCB) is critical to ensuring the reliability of electronic systems. However, the presence of complex circuit backgrounds and the small scale of defects often limit the precision and effectiveness of conventional inspection approaches. To address these challenges, this paper proposes FMW-YOLO, a lightweight and accurate detection framework based on YOLO11n. Specifically, a Frequency-Enhanced Channel-Transposed and Local Feature backbone network is developed to improve feature extraction. By designing a Dual-Frequency and Channel Attention Aggregation module and a Lightweight Edge-Gaussian Block, the original C3k2 structure is refined to suppress noise interference while preserving high-frequency details, thereby enhancing feature representation. Furthermore, a neck network incorporating a Multi-Scale Context-Aware Enhancement mechanism is constructed, in which an Attention-Integrated Feature Pyramid is employed to facilitate more effective cross-scale feature interaction. In addition, a Dilated Reparam Residual Module is embedded into the C3k2 structure to expand the receptive field without significantly increasing computational burden. Finally, Wise-IoU is adopted to optimize bounding box regression by assigning greater importance to anchors of moderate quality. Extensive experiments conducted on the HRIPCB and DeepPCB datasets demonstrate that FMW-YOLO improves mAP50 by 2.1% and 0.3%, respectively, while reducing the number of parameters by 23%. These results indicate that the proposed method achieves improved detection accuracy and demonstrates strong potential for practical industrial applications.

## 1. Introduction

With the continuous advancement of the Industry 4.0 paradigm, the electronics sector is steadily moving toward higher levels of intelligence and automation. PCBs, as a fundamental element of modern electronic systems, play a pivotal role in ensuring the stability and reliability of final products. However, defects on PCB surfaces are unavoidable during manufacturing, storage, and transportation processes. Even slight imperfections may adversely affect product performance and undermine overall quality, potentially leading to considerable economic losses. Consequently, the development of high-precision and robust PCB surface defect detection techniques has become increasingly critical in contemporary electronic manufacturing.

Traditional PCB surface defect detection methods primarily include manual inspection [1], electrical inspection [2] and Automated Optical Inspection (AOI) [3]. Manual inspection relies heavily on the expertise and concentration of human operators, making it prone to visual fatigue, which in turn leads to low efficiency and relatively high rates of missed detections and false alarms [1]. Compared with manual inspection, the electrical inspection improves detection accuracy; however, it requires direct physical contact with the PCB during the inspection process, which may cause secondary damage. Moreover, this approach depends on specialized equipment and fixtures, leading to increased costs and limited flexibility [2]. In recent years, AOI systems have been extensively adopted, offering improved detection accuracy and higher efficiency compared with manual inspection. Despite these benefits, AOI systems entail substantial equipment investment, and their performance is still affected by non-negligible false-positive and missed-detection rates [4].

With the continuous advancement of surface defect detection techniques, numerous studies have employed image processing-based methods, such as threshold segmentation [5] and edge detection [6]. While these methods can be effective in relatively simple scenarios, their performance degrades in PCB inspection tasks due to the presence of complex and cluttered backgrounds. In such cases, defect extraction largely depends on local image cues, which restricts robustness and limits generalization across varying conditions [1]. In addition, traditional machine learning-based methods, including decision trees [7], random forests [8], and support vector machines [9], have also been applied to PCB defect detection. However, their effectiveness is closely tied to the quality of handcrafted features and classifier design. In practice, these models often involve considerable computational overhead and exhibit limited inference speed, which hinders their deployment in real-time industrial environments.

In recent years, deep learning-based detection techniques have achieved remarkable progress across various domains, including industry [10,11], agriculture [12,13], transportation [14,15], etc. These approaches benefit from powerful feature representation capabilities and efficient inference processes. With the continued advancement of deep learning in PCB surface defect detection, existing detection algorithms can generally be categorized into two-stage and one-stage frameworks. Two-stage approaches, represented by R-CNN [16], Fast R-CNN [17], and Faster R-CNN [18], are known for their high detection accuracy. However, their high computational complexity, slow inference speed, and long processing time limit their applicability in scenarios with constrained computing resources. In contrast, one-stage methods, such as YOLO [19] and SSD [20], are designed to strike a more favorable balance between accuracy and efficiency. Although SSD improves detection accuracy and reduces false positives, it still involves a relatively large number of parameters and model size, which negatively affects detection speed [20]. In contrast, the YOLO family offers a more streamlined detection paradigm by directly learning discriminative features from raw input data [21] and performing object classification and localization simultaneously within a single forward pass. This unified design enables YOLO-based methods to achieve competitive accuracy while maintaining high inference efficiency.

Despite the significant progress made in deep learning-based research, YOLO-based PCB defect detection still faces notable challenges. These difficulties primarily arise from the complex background and the small size of surface defects, which make the detection process highly sensitive to noise. In particular, tiny defects typically present weak visual cues and prominent high-frequency characteristics, which are highly susceptible to degradation or loss during successive down sampling operations. Furthermore, many existing methods are constrained by limited receptive fields, reducing their ability to capture sufficient contextual information. In addition, commonly used feature fusion strategies typically assign equal weights to all channels, resulting in relatively coarse representations with limited discriminative capability. These factors restrict further improvements in both the accuracy and robustness of PCB defect detection.

To address the aforementioned challenges, this study presents a series of targeted enhancements to the feature extraction, feature fusion and loss function within the YOLO11n framework, resulting in the proposed FMW-YOLO model. Unlike existing approaches that primarily emphasize either feature enhancement or lightweight design, the proposed FMW-YOLO integrates frequency-aware representation, noise suppression, expansion of the receptive field and efficient multi-scale feature fusion within a unified framework, thereby achieving a more balanced and effective detection performance. The proposed framework not only improves detection accuracy but also reduces the number of parameters. These characteristics make the proposed method well suited for real-time PCB inspection in resource-constrained industrial environments. The main contributions of this paper are summarized as follows:

In the backbone network, a Frequency-Enhanced Channel-Transposed and Local Feature Network (FCT-LFNet) is proposed. We propose the Dual-Frequency and Channel Attention Aggregation (DFCAA) module and the Lightweight Edge-Gaussian Block (LEGB) module. The DFCAA module is used to recover high-frequency details and enhance the expressive capability of features, while the LEGB module effectively suppresses noise interference and enhances boundary representation.

In the neck network, a Multi-Scale Context-Aware Enhancement (MSCAE) network is designed. The Multi-Scale Feature Pyramid Network with Integrated Channel Attention (MSFPNICA) is proposed to enhance cross-scale feature interaction, thereby achieving effective multi-scale feature fusion. In addition, the Dilated Reparam Residual Module (DRRM) is designed in this network, thereby enlarging the receptive field.

Regarding loss function, to alleviate the issue that all predicted bounding boxes are treated with identical gradient updates during training, Wise-IoU is incorporated into the loss formulation. By adaptively down-weighting low-quality samples, this mechanism mitigates their adverse impact on optimization, thereby enhancing training stability and improving overall detection performance.

For validation, the effectiveness of the proposed method is validated through extensive experiments conducted on the HRIPCB and DeepPCB datasets, which are widely used benchmarks for PCB surface defect detection.

## 2. Related Work

This section reviews representative studies conducted by previous researchers on PCB surface defect detection based on machine learning and deep learning approaches.

### 2.1. Research Based on Traditional Machine Learning

Ma et al. [11] proposed an automated method for void detection PCB based on supervised machine learning. The method employs an end-to-end segmentation model to identify pixels belonging to void and integrated circuit (IC) regions in X-ray PCB images. The segmentation results are then used to calculate the percentage of voids within each IC, leading to improved detection accuracy. Liu et al. [22] introduced an effective lightweight defect detection network for edge scenarios, based on Knowledge Distillation (KD-Light Net), they designed a lightweight network using structural reparameterization and an improved KL divergence loss, addressing the requirements for real-time performance and model compactness in industrial applications.

### 2.2. Research Based on Transformer Architectures

Guan et al. [23] proposed a real-time defect detection model based on the Transformer architecture, PHDL-RTDETR, which integrates physics-guided and attention-based components to enhance performance, addressing challenges posed by small and complex defects as well as noise. Luo et al. [24] introduced a lightweight framework, Lite-DETR based on a real-time detection transformer, they designed a lightweight and efficient backbone network and an image feature augmentation module to overcome issues of structural complexity and poor generalization associated with transformer-based detectors.

### 2.3. Research Based on Convolutional Neural Networks

Zhang et al. [25] proposed a high-precision lightweight PCB defect detection network LDD-Net, which incorporates a novel lightweight feature extraction network and a multi-scale aggregation network, addressing the trade-off between accuracy and computational cost. Hu et al. [18] developed an improved Faster R-CNN-based network, employing ResNet50 with a Feature Pyramid Network (FPN) as the backbone for feature extraction, using GARPN to predict more accurate anchors, and integrating ShuffleNetV2 residual units to mitigate noise and reduce computational cost. Yu et al. [26] introduced a novel deep neural network with an adaptive key point localization network to tackle the challenge of irregular and small defects on PCB. Ran et al. [27] proposed a PCB defect detection algorithm based on the Single Shot Detector (SSD) framework, where they used multi-scale feature mapping to customize bounding boxes of different scales, small convolution kernels to predict classification results and bounding box information, and optimized the detection results through Non-Maximum Suppression (NMS), solving the problem of low robustness of existing traditional detection algorithms. Wu et al. [28] proposed a RAR-SSD that combined multi-scale PCB defect detection with an attention mechanism, and they also constructed a feature fusion module to efficiently fuse low-level feature information with high-level feature information, solving the challenge of PCB defects being too small to be identified.

### 2.4. Research Based on the YOLO Series

Tang et al. [29] proposed a lightweight PCB defect detection model, Light-PDD, based on YOLO v4, which used a pruned MobileNetV3 structure for feature extraction and an improved Cross-Stage Partial (CSP) structure for feature fusion, solving the problems of redundant parameters and slow inference speed. Yuan et al. [30] proposed an improved YOLO-HMC network based on YOLO v5, which can identify small-sized PCB defects more accurately and efficiently with fewer model parameters. Hou et al. [31] proposed a lightweight detection model efficient network for PCB based on YOLO v8; in addition, the use of multi-scale convolutional block attention module improved the sensitivity to different defects and solved the problem of balancing detection accuracy and speed. Tie et al. [32] proposed a lightweight surface defect detection model, LSKA-YOLOv8, based on YOLO v8n; this model used Kernel Warehouse Conv (KWConv) with low computational complexity to solve the problem of large computational complexity and difficult deployment. Wang et al. [33] proposed a YOLOX-MC-CA based on YOLOX; this network had an improved CSPDarknet and coordinated attention, which solved the problems of low detection efficiency, high memory consumption, and low sensitivity to small defects of traditional detection networks. Hou et al. [34] proposed an efficient path aggregation network in the cross-layer feature fusion stage, which adopted a prior-based adaptive fusion strategy to replace traditional feature fusion methods, thereby improving small object detection performance. Yu et al. [35] proposed a receptive field enhancement module to capture multi-scale pixel-level information and enlarge the receptive field, thereby improving the accuracy of small face detection. Yang et al. [36] introduced a receptive field channel attention convolution module to replace the standard convolutional block, enabling dynamic receptive field adjustment for improved feature capture across different scales.

Despite notable progress, existing PCB inspection methods remain limited in their feature representation capability. CNN- and Transformer-based models predominantly emphasize spatial dependency modeling via self-attention, yet fail to adequately capture fine-grained channel-wise information. This deficiency is particularly evident in the representation of high-frequency structures, such as edges and textures, which are prone to degradation. Although YOLO-based approaches introduce context-aware designs and enhanced feature fusion mechanisms to mitigate these issues, their effectiveness is still constrained. Most existing studies have been heavily biased toward spatial context and cross-scale interaction while largely neglecting frequency-domain modeling. This inherent limitation results in insufficient characterization of high-frequency details, especially under complex background conditions.

## 3. Materials and Methods

### 3.1. Overview of YOLO11

**Figure 1:**
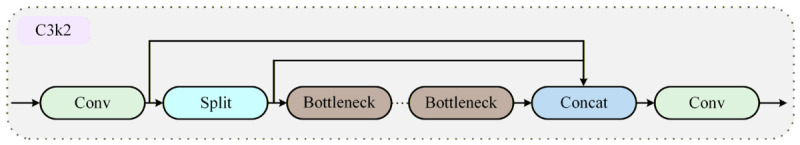
The C3k2 structure in YOLO11n.

The YOLO11 algorithm [37], proposed by the Ultralytics team, represents a recent advancement in the YOLO family of object detection models. As an end-to-end one-stage detection algorithm, it achieves a favorable balance between detection accuracy and inference efficiency, while also exhibiting improved generalization capability. Compared with previous versions such as YOLOv8, YOLO11 introduces significant optimizations in network architecture design, feature extraction efficiency and training strategies. YOLO11 provides five model variants: YOLO11n, YOLO11s, YOLO11m, YOLO11l, and YOLO11x. These variants differ in network depth, width, and the maximum number of channels, resulting in progressively increasing parameter sizes and computational costs [38]. The overall architecture of YOLO11 mainly consists of three components: a backbone for feature extraction, a neck for feature fusion, and a head for generating final detection results [39]. Among them, the C3k2 module, illustrated in Figure 1, serves as a key component that improves upon the traditional C3 structure to enhance feature extraction capability, particularly for complex and multi-scale scenarios. To achieve a balance between detection accuracy and computational efficiency, this study adopts YOLO11n as the baseline model.

### 3.2. Architecture of FMW-YOLO

**Figure 2:**
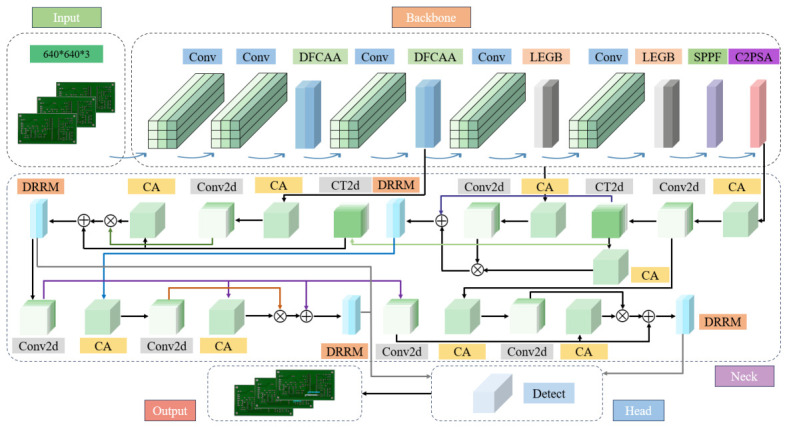
Overall framework of FMW-YOLO. CA represents channel attention and CT2d represents ConvTranspose2d.

The overall architecture of FMW-YOLO is illustrated in Figure 2. In the backbone network, a Dual-Frequency and Channel Attention Aggregation (DFCAA) module is proposed to effectively integrate high-frequency information derived from shallow features with the original representations, thereby enhancing the recovery of fine-grained details and improving feature expressiveness. Furthermore, a Lightweight Edge-Gaussian Block (LEGB) is designed to alleviate noise interference and enhance robustness under low-quality and low-contrast imaging conditions. Building upon these components, the Frequency-Enhanced Channel-Transposed and Local Feature Network (FCT-LFNet) is further constructed to achieve more comprehensive multi-scale feature extraction.

In the neck network, the Multi-Scale Feature Pyramid Network with Integrated Channel Attention (MSFPNICA) is constructed to adaptively recalibrate channel-wise fusion weights, enabling more discriminative feature aggregation. Furthermore, to expand the receptive field and capture richer contextual information, a Dilated Reparam Residual Module (DRRM) is designed. By integrating MSFPNICA with DRRM, the novel Multi-Scale Context-Aware Enhancement (MSCAE) network is developed, which promotes effective multi-scale feature integration and strengthens the overall representational capacity of the model.

Finally, the Wise-IoU loss function is introduced to alleviate the excessive competition among high-quality anchor boxes while mitigating the adverse gradients generated by low-quality samples [40].

### 3.3. Frequency-Enhanced Channel-Transposed and Local Feature Network

In PCB surface defect detection, feature extraction often exhibits limited capability in modeling channel-wise feature relationships, insufficient recovery of high-frequency details, and weak adaptability to defect contexts across multiple scales. Moreover, the complex background of PCB surfaces makes it difficult to accurately delineate defect boundaries under conditions of noise and low contrast, which negatively affects detection robustness. To address these challenges, a Frequency-Enhanced Channel-Transposed and Local Feature Network (FCT-LFNet) is proposed, which integrates multi-frequency channel enhancement with edge-aware Gaussian refinement. Specifically, a Dual-Frequency and Channel Attention Aggregation (DFCAA) module is designed to capture channel dependencies while jointly extracting low- and high-frequency components, thereby improving the representation of fine-grained details. Meanwhile, a Lightweight Edge-Gaussian Block (LEGB) is introduced to adaptively combine shallow edge information with deep Gaussian representations, enabling effective noise suppression and clearer boundary localization. The complementary integration of these two components significantly improves the model’s capability in detail recovery and boundary identification.

#### 3.3.1. Dual-Frequency and Channel Attention Aggregation

**Figure 3:**
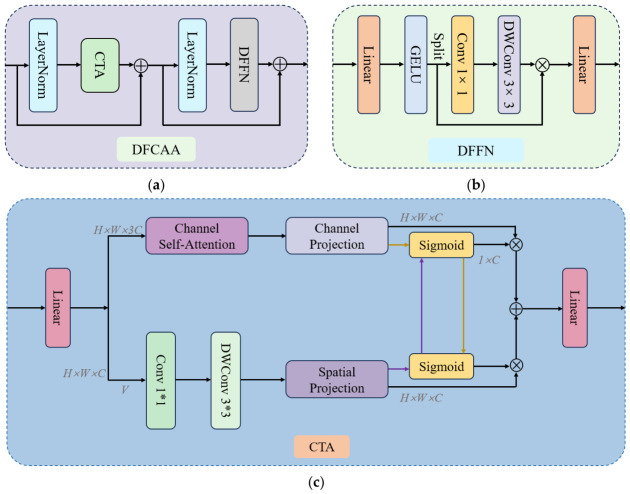
Overall architecture of the DFCAA. (**a**) DFCAA; (**b**) DFFN; (**c**) CTA.

While low-frequency components preserve global structure and semantic stability, the effective restoration of high-frequency information remains critical for accurate detail representation. Enhancing high-frequency responses not only strengthens inter-channel dependencies but also improves the fidelity of fine-grained feature reconstruction, thereby benefiting overall representation quality. To this end, a Dual-Frequency and Channel Attention Aggregation (DFCAA) module is proposed to refine selected C3k2 within the YOLO11n backbone. The original bottleneck units in C3k2 are replaced with the proposed DFCAA to enhance feature extraction capability. By incorporating channel-level self-attention and explicit frequency-aware modeling, DFCAA effectively addresses the limitations of conventional designs in capturing high-frequency details. The structure of DFCAA is illustrated in Figure 3a, where Channel Transposed Attention (CTA) and Dual-Frequency Feed-Forward Network (DFFN) serve as key components for feature transformation and enhancement.

The Channel Transposed Attention (CTA) is designed to perform self-attention operations along the channel dimension, enabling more effective modeling of inter-channel dependencies and overcoming the limitation of conventional attention mechanisms that predominantly emphasize spatial information. By leveraging channel-wise interactions, CTA facilitates the prioritization of informative features and enhances detail preservation during feature reconstruction. The structure of CTA is illustrated in Figure 3c. First, the input features are processed by dividing the input feature map into multiple channels. During the self-attention computation, the query (*Q_Z_*), key (*K_Z_*) and value (*V_Z_*) representations are generated to capture the relationships across different channels. The attention operation can be formulated as follows:

(1) $$F_{C-A}=SoftMax\left({\left(Q_{Z}\right)}^{T}K_{Z}/\alpha \right)\cdot V_{Z},$$

where α denotes a learnable temperature parameter used to adjust the scale of the dot-product operation, while *F_C-A_* refers to the feature obtained by performing self-attention computation along the channel dimension. Through the aforementioned self-attention calculation, CTA effectively models inter-channel dependencies, allowing the network to emphasize more informative channel responses during feature reconstruction. Subsequently, CTA reorganizes the connections between different attention heads to generate the channel attention feature *F_CA_*. To reduce computational cost, spatial and channel features are integrated only within the channel attention mechanism. For additional feature dimensions, the same projection strategy is adopted to obtain both attention features *F_C_*_1_ and *F_C_*_2_, as well as the spatial projection output *Y_S_*. These representations are then utilized for feature extraction and cross-domain weighting, and the overall computation can be formulated as follows:

(2) $$F_{CA}=F_{C1}\cdot f(Y_{S})+\mathrm{DWConv}(V_{Z})\cdot f(F_{C2}),$$

where *f*(·) represents the sigmoid activation function. Finally, the spatial attention feature weights are used to modulate the CA output features, while the CA features are inversely used to reweight the spatial attention features. This complementary interaction effectively integrates spatial attention features with channel attention features, thereby enhancing the final feature representation.

The Dual-Frequency Aggregation Feed-Forward Network (DFFN) is designed to enhance high-frequency representations for improved recovery of fine-grained details. Conventional attention mechanisms often exhibit a bias toward low-frequency components, which may result in the attenuation of high-frequency information. To mitigate this issue, DFFN explicitly models frequency decomposition, enabling effective detail enhancement while preserving global structural consistency. The architecture of DFFN is illustrated in Figure 3b. First, the input feature *F_CA_* is projected to *Y_in_* through a fully connected layer and then activated by the GELU function. Subsequently, a frequency gating mechanism is employed to separate the low- and high-frequencies for independent processing. The low-frequency information is retained to maintain global structural stability, whereas the high-frequency branch is refined using 1 × 1 convolution and depth-wise convolution (DWConv) to enhance local details. The calculation formula for frequency gating is:

(3) $$Y_{fg}=Y_{in}\cdot \mathrm{DWConv}(\mathrm{Conv}1\times 1(Y_{in})),$$

where *Y_fg_* is the result of element-by-element multiplication of the two features. This design enables effective integration of feature representations with high-frequency components from both branches. Through this dual-frequency information aggregation strategy, DFFN preserves the global structural information while preventing the loss of high-frequency components. By jointly aggregating low- and high-frequency information, the module further improves the restoration of fine-grained image details.

In summary, the DFCAA module incorporates both CTA and DFFN to strengthen high-frequency feature modeling, thereby improving the fidelity of fine-grained detail reconstruction.

#### 3.3.2. Lightweight Edge-Gaussian Block

Owing to the small size of PCB components and the dense distribution of pads, many defects frequently occur in pad regions where distinguishing defect boundaries from structural patterns becomes challenging. This significantly increases the difficulty of feature extraction. Moreover, noise interference further degrades edge clarity, leading to ambiguous object boundaries. To address these challenges, a Lightweight Edge-Gaussian Block (LEGB) is designed and incorporated into the last two C3k2 stages of the YOLO11n backbone, replacing the original bottleneck units. This proposed block employs an edge-Gaussian aggregation mechanism to effectively mitigate boundary ambiguity, suppress noise interference and enhance boundary representation as well as feature detail preservation, thereby improving the overall robustness of the model.

**Figure 4:**
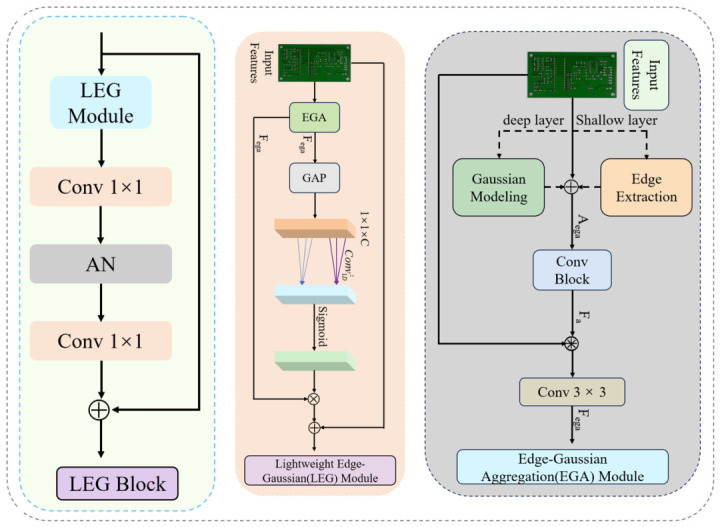
Architecture of the LEGB.

As shown in Figure 4, the proposed LEGB achieves a favorable balance between edge-aware information and global features, enabling the network to extract more discriminative representations even under noisy conditions and low-contrast scenarios. Specifically, the input feature *F_in_* is first processed by the Lightweight Edge-Gaussian Module (LEGM) to enhance low-quality feature representations. The resulting features are then fed into a 1 × 1 convolution layer, followed by an Activation–Normalization (AN) operation, yielding the output feature *F_mid_*:

(4) $$F_{mid}=\mathrm{AN}({\mathrm{Conv}}_{2D}^{1\times 1}(\mathrm{LEGM}(F_{in}))),$$

where ${Conv}_{2D}^{1\times 1}$ denotes a two-dimensional 1 × 1 convolution. Next, a second 1 × 1 convolution is applied to adjust the channel dimension to *C*. This is followed by normalization and a dropout operation with a rate of 0.1. Finally, the processed feature is added to the initial input via a residual connection, producing the output feature *F_out_*:

(5) $$F_{out}=F_{in}+\mathrm{Norm}(\mathrm{Drop}({\mathrm{Conv}}_{2D}^{1\times 1}(F_{mid}))).$$

The LEGM first applies an edge-Gaussian aggregation (EGA) module to the input features, producing an intermediate feature *F_ega_*. To further emphasize the more informative channels, the Efficient Channel Attention (ECA) strategy [41] is introduced. The formulation of this mechanism can be expressed as follows:

(6) $$F_{temp}=Sigmoid({\mathrm{Conv}}_{1D}^{z}(\mathrm{GAP}(F_{ega}))),$$

(7) $$F_{o}=\mathrm{Norm}((F_{temp}⊗F_{ega})+F_{in}),$$

where ${Conv}_{1D}^{z}$ indicates an adaptive convolution along one dimension whose kernel size z is proportionally related to the number of channels C; GAP refers to channel-wise global average pooling; ⊗ indicates the element-wise multiplication operation; *F_temp_* denotes the output feature after the Sigmoid function; and *F_o_* denotes the output feature generated by the LEGM.

The EGA module introduces an edge-Gaussian aggregation mechanism that adaptively fuses edge cues with Gaussian modeling responses through a weighted integration strategy, thereby enhancing feature representation. Furthermore, the module employs a stage-wise selection strategy for the input features *F_in_*, where shallow layers are primarily responsible for edge feature extraction, while deeper layers focus on Gaussian modeling. The output feature obtained through EGA is denoted as *A_ega_*:

(8) $$A_{ega}=\left\{\begin{array}{l} A_{edga}(F_{in}),\;\;\;\;\;\;\;\;\;\;\;\;\;\;shallow\;\;layer,\\ A_{gauss}(F_{in}),\;\;\;\;\;\;\;\;\;\;\;\;\;deep\;\;layer. \end{array}\right.$$

where *A_edga_* denotes the edge feature extraction operation, and *A_gauss_* denotes the Gaussian modeling operation. The obtained *A_ega_* is combined with the input *F_in_* and subsequently refined through a three-layer convolutional block, resulting in the enhanced feature:

(9) $$\begin{array}{l} F_{temp}={\mathrm{Conv}}_{2D}^{3\times 3}(\mathrm{AN}({\mathrm{Conv}}_{2D}^{1\times 1}(F_{in}+A_{ega}(F_{in})))),\\ ConvBlock(F_{in})=\mathrm{Norm}({\mathrm{Conv}}_{2D}^{1\times 1}(\mathrm{AN}(F_{temp}))), \end{array}$$

where ${Conv}_{2D}^{3\times 3}$ denotes a two-dimensional 3 × 3 convolution. Finally, the convolutional block output *F_a_* is combined with input *F_in_* through element-wise multiplication and addition operations, and then through 3 × 3 convolution to obtain the enhanced feature:

(10) $$F_{ega}={\mathrm{Conv}}_{2D}^{3\times 3}((F_{in}⊗F_{a})⊕F_{in}),$$

where ⊕ denotes the element-by-element addition operation.

In summary, the LEGB preserves fine-grained boundary information through the edge-Gaussian aggregation mechanism, effectively suppresses the adverse effects of noise, and improves the robustness of detection in complex background scenarios.

### 3.4. Multi-Scale Context-Aware Enhancement

During inference, defects often exhibit diverse scales and sparsely distributed, which impose higher requirements on contextual modeling capabilities. To this end, a Multi-Scale Context-Aware Enhancement (MSCAE) network is proposed to improve feature interaction efficiency and contextual information. The proposed network consists of two key parts: Multi-Scale Feature Pyramid Network with Integrated Channel Attention (MSFPNICA) and the Dilated Reparam Residual Module (DRRM). Specifically, MSFPNICA enhances cross-scale feature interaction and improves the preservation of small-object information through channel-adaptive weighting and bidirectional selective fusion. Meanwhile, DRRM expands the receptive field using dilated convolution and reparameterization strategies, enabling more effective modeling of sparse features and contextual dependencies. By integrating these two complementary modules, the proposed framework strengthens global semantic modeling while maintaining fine-grained detail recovery, thereby producing more discriminative feature representations for subsequent defect localization and classification in the detection head.

#### 3.4.1. Multi-Scale Feature Pyramid Network with Integrated Channel Attention

**Figure 5:**
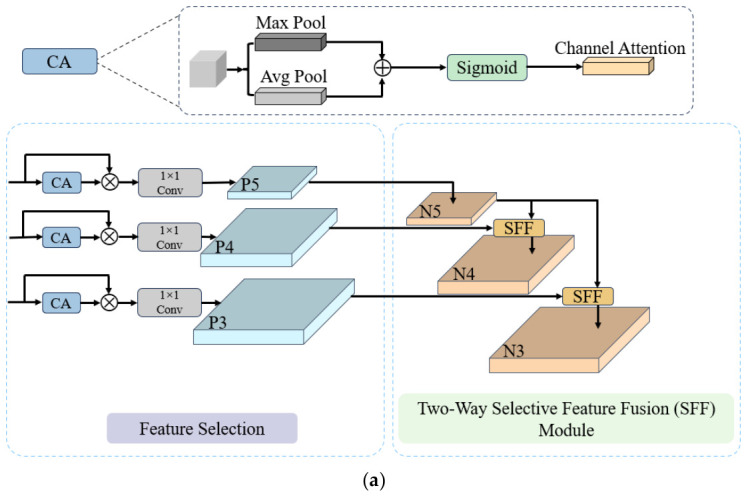

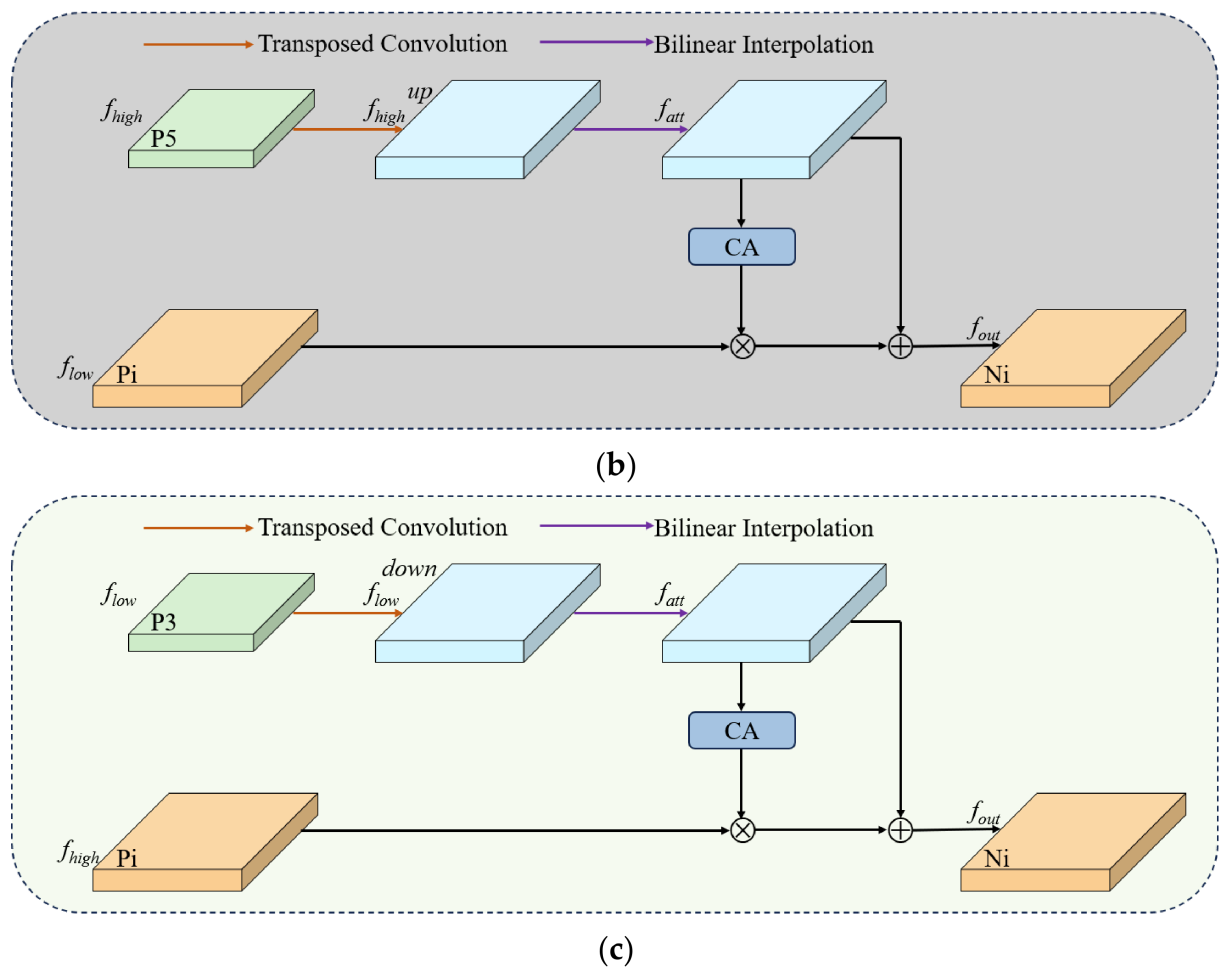
Overall architecture of the MSFPNICA. (**a**) Architecture of the MSFPNICA; (**b**) the top-down SFF; (**c**) the down-up SFF.N3, N4 and N5 represent the feature maps at the corresponding levels in the bottom-up path.

In PCB surface defect detection, Feature Pyramid Networks (FPNs) [42] are widely used for multi-scale feature fusion. By employing a top-down pathway with lateral connections, FPNs integrate high-level semantic information with low-level spatial details, thereby improving multi-scale representation capability. However, standard FPN architectures and their variants still suffer from several limitations. First, they lack fine-grained modeling in the channel dimension. Most existing designs rely on simple element-wise addition for feature fusion, implicitly treating all channels as equally important. Under complex industrial scenarios, such an assumption may introduce redundant or less informative features, thereby degrading detection performance. Moreover, the overly simplified fusion strategy makes it difficult to effectively exploit complementary information across different feature scales. To address these issues, we propose a Multi-Scale Feature Pyramid Network with Integrated Channel Attention (MSFPNICA) architecture, which incorporates the channel attention (CA) mechanism to enhance feature modeling. As shown in Figure 5a, the proposed architecture enhances channel-wise feature modeling and improves cross-scale feature interaction, leading to more effective multi-scale feature fusion.

We adopt a dual fusion strategy with bidirectional feature transfer, guided by the CA mechanism. This architecture provides significant advantages in cross-scale feature interaction, channel-selective modeling, and the preservation of small objects. Before feature fusion, CA is employed to adaptively reweight the input features *F*, enabling the network to emphasize more informative channels while suppressing less relevant ones. The resulting output feature is denoted as *F_ca_*:

(11) $$F_{ca}=Sigmoid(F_{\max}⊕F_{avg}),$$

where *F_max_* and *F_avg_* represent maximum pooling and global average pooling. This process highlights significant channels and suppresses redundant information. During the feature selection stage, at each layer (P3, P4, P5), the CA-enhanced features are adaptively reweighted in a channel-wise manner. Subsequently, a 1 × 1 convolution is applied to perform dimensionality reduction:

(12) $$P_{i}={\mathrm{Conv}}_{1\times 1}(F_{ca}^{\left(i\right)}⊗F),\;\;\;\;\;\;i\in \left\{3,\;4,\;5\right\}.$$

During the stages of dual-feature fusion and bidirectional feature propagation, the Selective Feature Fusion (SFF) submodule is employed for feature fusion and information transfer. The top-down and down-up structures are shown in Figure 5b,c. To avoid information loss caused by simple element-wise addition, a hybrid fusion strategy combining multiplicative modulation and additive compensation is adopted. In the top-down pathway, high-level semantic features are used as guiding signals to modulate the fusion process, enabling the preservation of essential semantic information from low-level representations. Specifically, both high-level and low-level features are taken as inputs *f*. The high-level features are first expanded via transposed convolution and subsequently up sampled using bilinear interpolation to align their spatial resolution with that of the low-level features. Afterwards, the CA mechanism is applied to transform the high-level features into attention weights, thereby enabling more refined feature integration. The final output *f_out_* can be expressed as:

(13) $$f_{out}=(f_{low}⊗\mathrm{CA}(\mathrm{Inter}(\mathrm{TConv}(f_{high}))))⊕(\mathrm{Inter}(\mathrm{TConv}(f_{high}))),$$

where Inter represents bilinear interpolation; TConv represents transposed convolution; the variables *f_low_* and *f_high_* denote the low-level and high-level feature maps, respectively; and CA indicates the channel attention mechanism. The down-up propagation follows a symmetrical process, with its final output *f_out_* expressed as:

(14) $$f_{out}=(f_{high}⊗\mathrm{CA}(\mathrm{Inter}(\mathrm{TConv}(f_{low}))))⊕(\mathrm{Inter}(\mathrm{TConv}(f_{low}))).$$

In summary, the MSFPNICA structure solves the limitation of the channel-equality assumption through the CA mechanism. The proposed dual fusion strategy, which integrates multiplicative modulation with additive compensation, achieves a balance between feature selection and information preservation, thereby enhancing the efficiency of multi-scale feature interaction within the model.

#### 3.4.2. Dilated Reparam Residual Module

**Figure 6:**
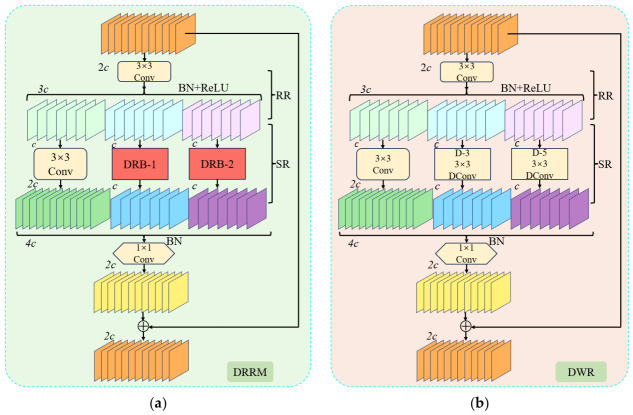

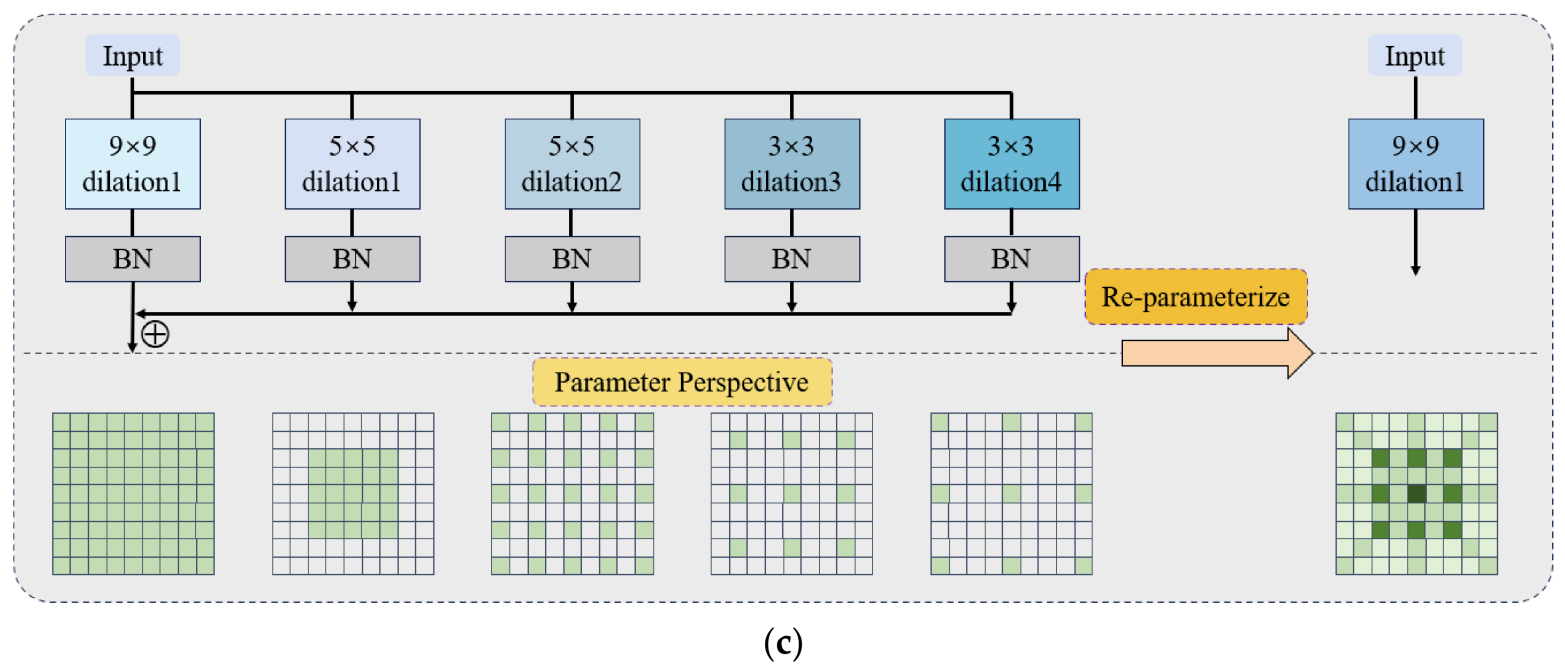
Overall architecture of the DRRM. (**a**) DRRM; (**b**) DWR; (**c**) DRB. Note: Conv denotes standard convolution; DConv refers to depth-wise convolution; D-n indicates a convolution operation employing a dilation factor of n; ⊕ represents element-wise addition; and *c* represents the cardinality of feature channels.

Traditional convolutional networks typically rely on small kernel sizes, which lead to limited receptive fields and insufficient contextual information capture, thereby constraining effective feature fusion. Moreover, due to the highly imbalanced distribution of PCB defect patterns, conventional models often struggle to adequately capture sparse structures, leading to increased false positives and missed detections. To tackle these challenges, we further enhance the MSFPNICA and propose the Dilated Reparam Residual Module (DRRM), as shown in Figure 6a. This module replaces the original bottleneck units within the C3k2 in the neck network, thereby expanding the receptive field and strengthening the model’s ability to capture complex spatial features.

In the YOLO11n architecture, the C3k2 module typically employs 3 × 3 convolutions. However, the limited receptive field of small kernels restricts its ability to capture sufficient contextual information. To address this limitation, the Dilation-Wise Residual (DWR) module is introduced to expand the receptive field by incorporating convolutions with multiple dilation rates, thereby enabling more comprehensive contextual modeling over a wider spatial range. The DWR adopts a two-step design comprising Region Residualization (RR)–Semantic Residualization (SR), as shown in Figure 6b. Each layer contains multiple dilated convolution branches with different dilation rates (e.g.,1, 3, 5, etc.). In the RR stage, convolution, ReLU activation, and batch normalization (BN) are sequentially applied to generate feature maps with diverse spatial representations. Subsequently, the SR stage processes these regional feature maps using dilated depth-wise convolutions with different dilation rates, allowing the model to capture multi-scale contextual dependencies. Each dilated branch operates on a distinct receptive field, thereby improving the model’s ability to perceive multi-scale features. The outputs of the dilated convolutions are incorporated into the original input through residual connection, which enhances information propagation and alleviates the gradient vanishing problem. By fusing these multi-scale features, the DWR effectively strengthens the capability of contextual information extraction, which has proven beneficial in dense prediction tasks such as semantic segmentation.

Due to the complex spatial characteristics of PCB defects, stronger feature representation capability is required. However, the aforementioned improvements still exhibit limited ability in capturing sparse patterns and may inevitably introduce additional computational overheads. To address these issues, we further enhance the DWR by incorporating the Dilated Reparam Block (DRB), which replaces the original 3 × 3 convolutions with parallel branches using dilation rates of 3 and 5. The architecture of the DRB module is illustrated in Figure 6c. The DRB adopts multiple small convolution kernels arranged in parallel, each operating with a different dilation rate to capture multi-scale spatial information. Dilated convolution introduces spacing between kernels to expand the receptive field without changing the kernel size. The outputs of these parallel convolutions are subsequently adaptively weighted and fused, followed by structural reparameterization to merge them into an equivalent large convolutional kernel. This strategy enables DRB to achieve the advantages of large-kernel convolution while avoiding additional computational burden. To evaluate the impact of integrating the DRB module on computational efficiency, frames per second (FPS) are compared in the experimental section. This analysis aims to demonstrate that the proposed structural design reduces the computational cost during the inference stage. Consequently, it improves the modeling of sparse spatial patterns, enhances receptive field expansion efficiency, and facilitates effective global feature extraction. By integrating these two approaches, the Dilated Reparam Residual Module (DRRM) is constructed. Specifically, DRRM first applies a standard 3 × 3 convolution for initial feature extraction, after which the feature maps are divided into three groups. Different processing strategies are applied to these groups to enhance the extraction of multi-scale semantic information. One group is processed using a 3 × 3 convolution, while the other two groups are processed by DRB modules with different receptive field sizes. Through multiple parallel dilated convolution branches, the DRB improves the ability of large convolution kernels to capture sparse patterns and obtain richer contextual information without increasing computational complexity. Finally, multi-level feature fusion is performed across different branches and interaction paths, thereby improving the detection capability for small objects and fine-grained details. By letting the input feature be denoted as *z*, the DRRM can be expressed as follows:

(15) $$F_{CBR}(z)=\mathrm{ReLU}(\mathrm{BN}({\mathrm{Conv}}_{3\times 3}(z))),$$

(16) $$F_{DD}(z)=\Gamma \left\{{\mathrm{DConv}}_{3\times 3}(F_{CBR}(z),1),\mathrm{DRB}((F_{CBR}(z))\right\},$$

(17) $$F_{out}=\mathrm{BN}({\mathrm{Conv}}_{1\times 1}(F_{DD}(z)))+z,$$

where ReLU denotes the activation function; Conv_3×3_(z) represents the standard 3 × 3 convolution; DConv_3×3_ indicates the 3 × 3 dilated convolution; DRB refers to the Dilated Reparam Block; Conv_1×1_ corresponds to the 1 × 1 pointwise convolution; Γ signifies the feature map concatenation operation; *F_CRB_*(*z*) denotes the feature map output after processing by ReLU, batch normalization and convolution operations; *F_DD_*(*z*) represents the feature map generated through DConv and DRB module processing; and *F_out_* indicates the final output feature.

In summary, the DRRM effectively expands the receptive field, improving the precision and efficiency of semantic segmentation. It enhances the modeling of sparse patterns and facilitates multi-scale information extraction, thereby strengthening the overall representational capacity of the network. Consequently, the proposed module effectively reduces the rates of missed detections and false positives.

### 3.5. Wise-IoU Loss Function

Traditional Intersection over Union (IoU)-based loss functions, such as SIoU, GIoU, DIoU and CIoU, exhibit certain limitations when handling low-quality prediction boxes. On the one hand, samples with low IoU values tend to introduce unstable gradient signals during the training process. On the other hand, these methods apply the same gradient update strategy to all prediction boxes, which prevents the model from effectively emphasizing high-quality predictions and consequently restricts further improvements in detection accuracy. To overcome these issues, the Wise-IoU loss function is introduced as the bounding box regression loss [40]. The parameters are set as ratio = 0.7, d = 0.0, and u = 0.95. This configuration adjusts the contribution of samples according to their IoU quality, thereby alleviating the impact of low-quality predictions and promoting more stable optimization. This approach incorporates a dynamic nonlinear weighting mechanism that assigns adaptive penalties to predictions of varying quality, effectively reducing the influence of low-quality samples while amplifying the contribution of high-quality ones during optimization. As a result, the proposed strategy improves training stability and enhances the robustness of the model. The traditional loss function can be expressed as follows:

(18) $$L_{IoU}=1-IoU(q_{p},q_{t}),$$

where *q_p_* is the predicted box and *q_t_* is the true box. The Wise-IoU is formulated as follows:

(19) $$L_{Wise-IoU}=1-IoU(q_{p},q_{t})+R_{W}(q_{p},q_{t}),$$

where *R_W_*(*q_p_, q_t_*) is the Wise penalty term, which is used to adaptively adjust the sample weight, and its form is defined as:

(20) $$R_{W}(q_{p},q_{t})=\alpha \cdot (1-e^{-\beta \cdot IoU}),$$

where *α* and *β* are adjustment parameters. When the IoU value is low, the penalty term increases accordingly, thereby weakening the gradient effect of low-quality samples during training. When the IoU value is high, the penalty term gradually approaches zero, enabling the model to place greater emphasis on optimizing high-quality prediction boxes.

In summary, the incorporation of Wise-IoU loss effectively alleviates the adverse influence of low-quality samples, leading to more stable optimization during training. Meanwhile, by assigning greater emphasis to moderately qualified anchor boxes, the model is able to better approximate the overall data distribution.

## 4. Results

### 4.1. Experimental Configuration Experimental Parameters and Experimental Configuration

The training configuration of FMW-YOLO is summarized as follows: the input image size is 640 × 640; each batch size inputs 32 images; the number of epochs is 300; the weight decay is 0.0005; the initial learning rate is 0.01; the momentum is 0.937; the random seed is set to 0; and the optimizer is SGD. The training process follows the default YOLO augmentation strategy, including Mosaic augmentation, random horizontal flipping, HSV-based color jittering, random scaling, and translation. The experimental configuration is presented in Table 1.

### 4.2. Dataset Description

To validate the effectiveness of FMW-YOLO and its generalizability across different datasets, experiments are conducted on the HRIPCB and DeepPCB datasets for testing. Following standard practice in deep learning, each dataset is divided into training, validation, and test sets at a ratio of 8:1:1 [38], and the split is fixed across all experiments.

#### 4.2.1. HRIPCB

**Figure 7:**
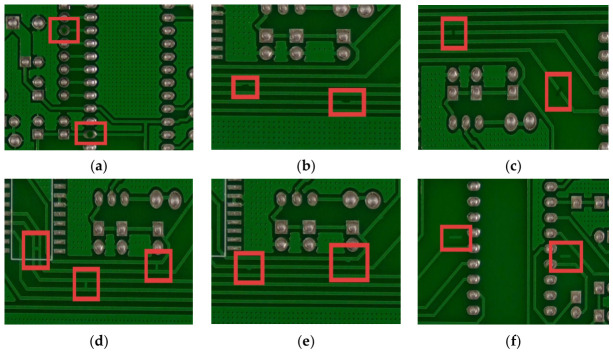
Defects on the HRIPCB: (**a**) Mh; (**b**) Mb; (**c**) Oc; (**d**) Sh; (**e**) Sp; (**f**) Sc.The red boxes indicate the specific locations where various defects are located.

The first dataset used in this study is the HRIPCB dataset [43], which was publicly released by the Intelligent Robotics Open Laboratory of Peking University. It comprises 693 images and covers six representative defect categories [32], including missing_hole (Mh), mouse_bite (Mb), open_circuit (Oc), short (Sh), spur (Sp), and spurious_copper (Sc), as shown in Figure 7.

#### 4.2.2. DeepPCB

To evaluate the generalization ability of the proposed method, the second dataset used in this study is DeepPCB [26], a public PCB defect detection dataset created by the Institute of Image Processing and Pattern Recognition at Shanghai Jiao Tong University. It includes 1500 pictures with the same defect types as HRIPCB. This dataset uses template matching to ensure image alignment, thereby reducing the need for additional preprocessing.

### 4.3. Experimental Evaluation Metrics

Common evaluation metrics in object detection tasks include recall, precision, average precision (AP) and mean average precision (mAP50) [23], the specific expressions are defined as follows:

(21) $$\mathrm{recall}=\frac{TP}{TP+FN},$$

(22) $$\mathrm{Precision}=\frac{TP}{TP+FP},$$

(23) $$\mathrm{AP}=\int_{0}^{1}P(r)dr,$$

(24) $$\mathrm{mAP}=\frac{1}{n+1}\sum_{i=1}^{n}{\mathrm{AP}}_{i},$$

where *TP* indicates a true positive; *FN* indicates a false negative; *FP* indicates a false positive; and n denotes the number of defect types.

### 4.4. Experimental Results

**Figure 8:**
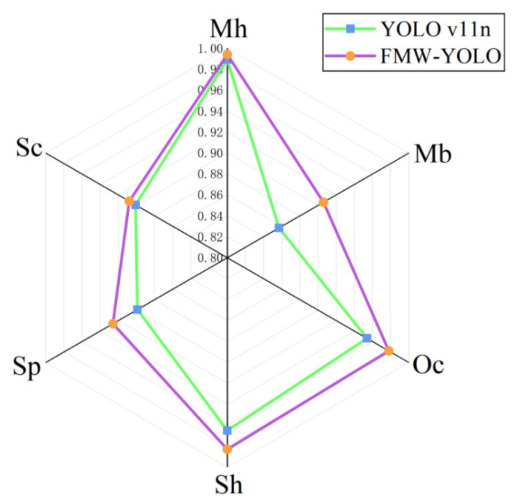
Comparison of mAP50 for different defect types.

The proposed model is evaluated on the HRIPCB dataset. Table 2 shows a comparison of performance metrics between the baseline YOLO11n and the proposed FMW-YOLO across different defect categories. Although a slight decrease in precision is observed for the Mb category, the corresponding recall is improved, accompanied by an increase in mAP50. A more intuitive comparison of mAP50 for different defect types is shown in Figure 8, which shows that the mAP50 values have improved for all defect categories. The results demonstrate that FMW-YOLO achieves effective and reliable performance for PCB defect detection tasks.

To assess the efficiency of the proposed model in practical scenarios, its inference performance is evaluated in terms of latency, frames per second (FPS), and model size. The corresponding results are presented in Table 3. Compared with the baseline, FMW-YOLO reduces the inference latency by 0.4 MS, achieves an approximate 25% increase in FPS, and decreases the model size by about 1 MB. These results indicate that the proposed method improves computational efficiency and reduces resource consumption while maintaining detection performance, suggesting its potential for practical industrial deployment.

### 4.5. Experiments Discussion

To verify the superiority of the proposed modules, comparative experiments are conducted on the HRIPCB dataset. The comparisons included the baseline YOLO11n, several representative backbone and neck architectures, as well as different IoU-based loss functions. In addition, the models developed in this study are examined under identical experimental settings to ensure a fair comparison. The results of these experiments further demonstrate the effectiveness and reliability of the proposed model.

#### 4.5.1. Backbone Comparison Experiments

To demonstrate the advantages of FCT-LFNet, comparative experiments are conducted by integrating several representative backbone networks into the YOLO11n framework, including VanillaNet [44], Unireplknet [45], Swin Transformer [46], STaRNet [47], and HGNetV2 [48], as presented in Table 4. The experimental results show that FCT-LFNet achieves superior performance across multiple evaluation metrics. Specifically, FCT-LFNet achieves an mAP50 of 93.7%, representing an improvement of 0.9 percentage points over YOLO11n, while maintaining a precision of 96.4% and a recall of 89.9%. Although its GFLOPs and parameter counts show a slight increase compared with YOLO11n, STaRNet and HGNetV2, the overall computational complexity remains lower than that of several other comparison networks, while delivering notable gains in detection performance.

#### 4.5.2. Neck Comparison Experiments

To evaluate the effectiveness of the MSCAE network, comparative experiments are conducted by integrating several representative methods into the YOLO11n framework, including GDSAFusion [49], SDI [50], Gold-YOLO [51], GLSA [52], and CSFCN [53], as shown in Table 5. The results show that MSCAE achieves competitive performance across multiple evaluation metrics. Specifically, MSCAE attains an mAP50 of 93.6%, representing an improvement of 0.8 percentage points over YOLO11n, while maintaining a precision of 94.3% and a recall of 89.3%. Notably, compared with YOLO11n, the proposed network reduces both parameters count and GFLOPs, leading to improved computational efficiency. Although GLSA achieves comparable average precision, its parameter counts and GFLOPs are 87.9% and 45.8% higher than those of MSCAE. Compared with other competing methods, MSCAE demonstrates clear advantages in precision, recall, average precision and computational complexity. These experimental results demonstrate that the proposed MSCAE achieves excellent performance in precision, recall and mAP50, while maintaining low computational complexity.

#### 4.5.3. IoU Comparison Experiments

To highlight the advantages of Wise-IoU, comparative experiments are conducted between Wise-IoU and other loss functions within the FMW-YOLO framework. The results presented in Table 6 indicate that Wise-IoU consistently outperformed the alternative loss formulations.

#### 4.5.4. Comparison of Different Models

**Figure 9:**
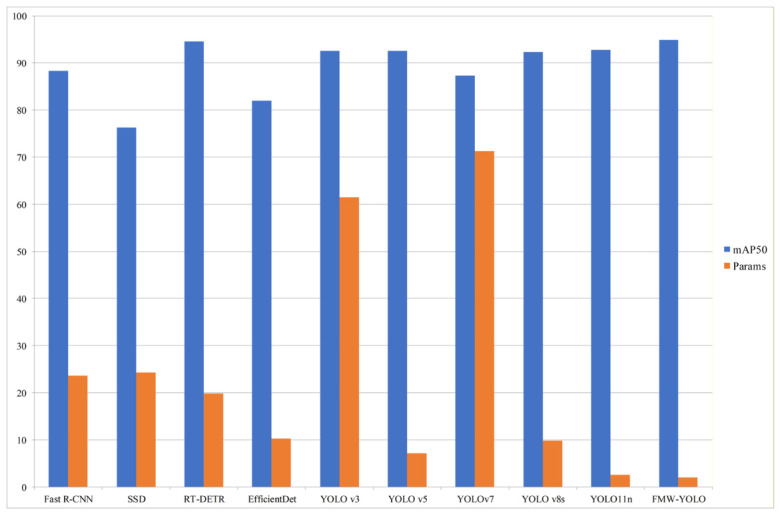
Comparison of mAP50 and param counts among different models.

To evaluate the overall performance of FMW-YOLO, comparative experiments are conducted between FMW-YOLO and several mainstream models on the HRIPCB dataset, as shown in Table 7. The proposed FMW-YOLO achieves an mAP50 of 94.9% and an mAP50-95 of 50.0%, significantly outperforming the other compared models. The comparison of mAP50 and the number of parameters among different models is illustrated in Figure 9. It can be clearly observed that FMW-YOLO achieves the highest detection accuracy while maintaining the lowest parameter count. This demonstrates that the proposed method attains a superior balance between performance and model complexity. Overall, FMW-YOLO not only improves detection accuracy but also reduces computational cost, all while effectively decreasing the rates of false detections and missed detections.

#### 4.5.5. Ablation Experiments

**Figure 10:**
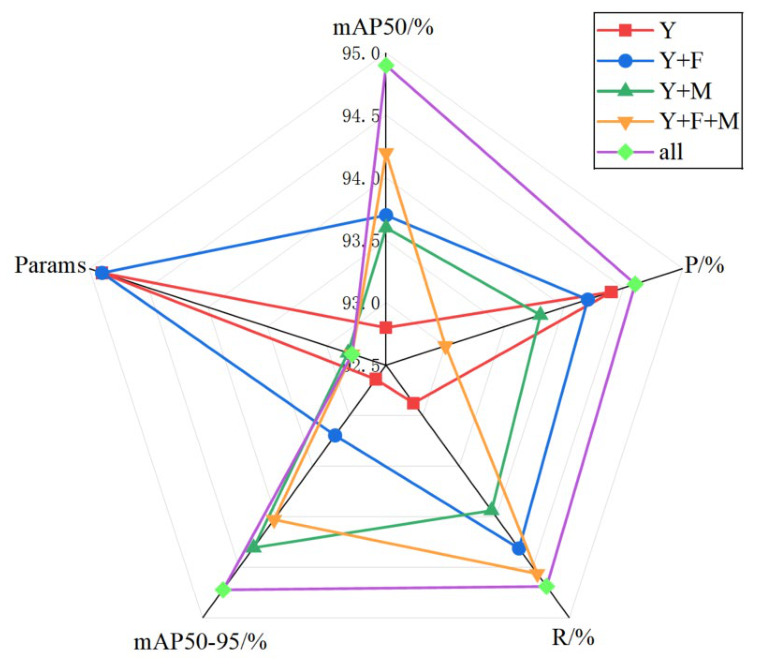
Visualization of ablation experiment results.

To provide a clearer assessment of the contributions of FCT-LFNet (F), MSCAE (M), and Wise-IoU (W), an ablation study is conducted on the YOLO11n baseline. Starting from the original model, modifications are progressively introduced to the backbone, the neck, their combination, and, finally, the complete framework. The results are shown in Table 8. Relative to the baseline, each modification leads to performance improvements to varying degrees. Specifically, the baseline YOLO11n achieves an mAP50 of 92.8% and an mAP50-95 of 48.5% with 2.57 M parameters. After introducing FCT-LFNet, the mAP50 increases by 0.9% and the mAP50-95 improves by 0.4%, which can be attributed to enhanced high-frequency detail recovery, improved noise suppression, and stronger feature extraction capability. After incorporating MSCAE, the mAP50 increases by 0.8% and the mAP50-95 improves by 1.2%, while the number of parameters decreases by 22.6%, making the model more lightweight. These gains result from more effective contextual modeling and enhanced cross-scale feature fusion. When both FCT-LFNet and MSCAE are introduced simultaneously, the mAP50 improves by 1.4%, the mAP50-95 improves by 1.0%, and the number of parameters decreases by 23%, which effectively reduces computational cost and resource requirements. Furthermore, when FCT-LFNet, MSCAE, and Wise-IoU are all integrated, the mAP50 improves by 2.1% and the mAP50-95 improves by 1.5%, while the number of parameters remains reduced by 23%. Wise-IoU assigns different gradient update strategies to prediction boxes of varying quality, thereby further improving the defect detection performance of FMW-YOLO. Figure 10 shows a clearer visualization of the impact of each improvement on the evaluation metrics. Overall, the results demonstrate that each proposed component contributes positively to enhancing feature extraction and feature fusion capabilities.

### 4.6. Inference Efficiency Analysis of DRRM

To examine whether the integration of DRB reduces the computational burden during inference, a comparison of Frames Per Second (FPS) is conducted. Based on the YOLO11n baseline, DWR, DRB and DRRM are sequentially incorporated for evaluation. The corresponding FPS values are measured under identical settings, and the results are summarized in Table 9.

The results indicate that the integrated DRRM achieves the highest inference speed among all configurations. Specifically, compared with the baseline model, the proposed approach achieves an improvement of approximately 43%. In addition, relative to the DWR-only variant, the inference speed increases by about 293%. These findings suggest that the proposed module enhances detection efficiency while maintaining low computational complexity, further highlighting the effectiveness of structural reparameterization in improving inference performance.

### 4.7. Visualization Experiments

**Figure 11:**
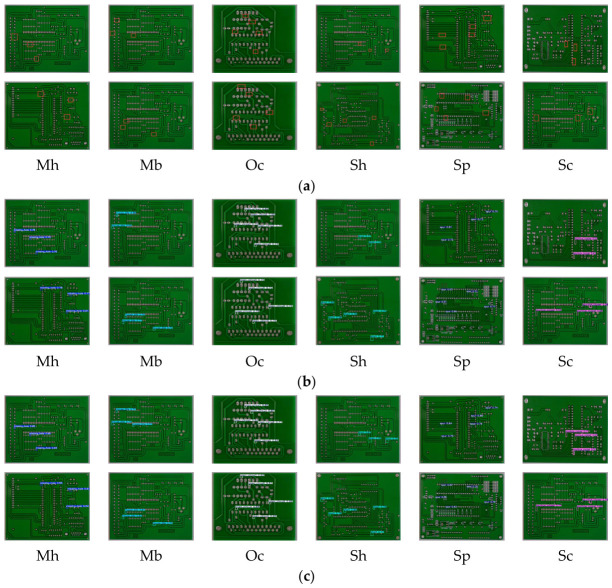
Visualization results on the HRIPCB. (**a**) Original Images; (**b**) YOLO11n; (**c**) FMW-YOLO. The red boxes in (**a**) indicates the specific locations where various defects are located.

To further validate the effectiveness of FMW-YOLO and provide a more intuitive comparison with YOLO11n, visualization experiments are conducted on the HRIPCB dataset. Representative images containing six defect categories are randomly selected for analysis, as illustrated in Figure 11. Compared with the baseline, FMW-YOLO exhibits improved detection performance, characterized by fewer missed detections and false positives, as well as enhanced localization accuracy. These results further confirm the reliability and effectiveness of the proposed method.

### 4.8. Generalization Experiments

**Figure 12:**
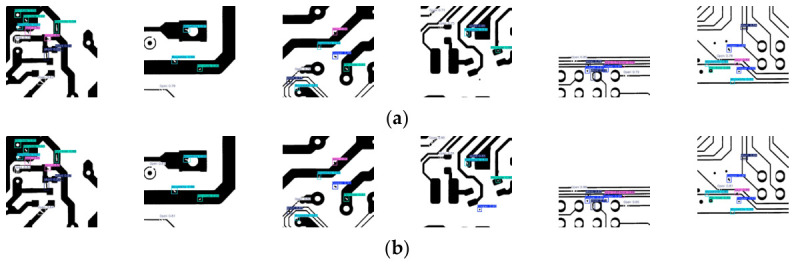
Visualization results on the DeepPCB. (**a**) YOLO11n; (**b**) FMW-YOLO.

To further demonstrate the generalization capability of FMW-YOLO, additional experiments are conducted on the DeepPCB dataset, accompanied by qualitative visualization analysis. The quantitative results are presented in Table 10, and the corresponding visualizations are shown in Figure 12. The results indicate that although the precision of FMW-YOLO slightly decreases for the Mh and Oc defect categories, the recall for these categories shows improvement. Overall, the model achieves higher precision, recall, and mAP50 compared with the baseline. Furthermore, a comparison evaluation between FMW-YOLO and other models is provided in Table 11, further supporting the effectiveness and practical applicability of the proposed approach.

Given that the performance gain on the DeepPCB dataset is relatively limited, additional repeated experiments are conducted to examine the reliability of the results. Specifically, with all other experimental settings kept identical, the model is trained using different random seeds (0, 42, 188, 1666, and 3407), and the corresponding statistics are summarized in Table 12 in terms of mean and standard deviation. As shown in Table 12, the proposed method consistently outperforms the baseline in terms of mAP50, while the variation across different runs remains small. The relatively low standard deviation suggests that the observed improvement is stable, rather than arising from randomness during training.

## 5. Discussion and Conclusions

This paper proposes FMW-YOLO, a frequency-domain enhanced and multi-scale context-aware detection model based on YOLO11n. The proposed model achieves superior detection performance for PCB images characterized by complex background lines as well as small and irregular defects.

First, FCT-LFNet is designed by integrating DFCAA and LEGB. DFCAA introduces frequency-domain features, while LEGB adopts an edge-Gaussian aggregation mechanism. The combination of these two components effectively restores high-frequency details and suppresses noise interference, thereby enhancing the model’s feature extraction capability. Second, MSCAE consisting of MSFPNICA and DRRM is constructed. MSFPNICA incorporates a channel attention mechanism to enhance feature interaction, while DRRM expands the receptive field through convolutions with different dilation rates. The integration of these two modules improves the efficiency of cross-scale feature fusion and enhances the robustness of the model under complex background conditions. Finally, Wise-IoU is introduced to mitigate the negative impact of low-quality samples through an adaptive gradient balancing mechanism, thereby stabilizing the detection process and improving localization accuracy.

Since Sc and Sp exhibit similar shapes, distinguishing between these poses certain challenges for accurate detection. Specifically, Sc occurs outside the PCB, whereas Sp appears on the PCB. In future work, we will focus on further improving the detection performance for complex defect categories such as Sc and Sp, enabling more accurate discrimination between defects located on the circuit and those occurring outside the circuit. Additionally, we plan to explore the integration of the proposed framework with Transformer-based hybrid architecture to broaden its applicability across diverse manufacturing scenarios.

## Figures and Tables

**Table 1:** Experimental configuration.

Configuration	Version
Python	3.10.15
Operating system	Ubuntu 22.04
CUDA	12.1 parallel computing platform
Pytorch	2.3.0
GPU	RTX 4090
CPU	AMD EPYC 7352
RAM	60 GB

**Table 2:** Comparison of YOLO11n and FMW-YOLO for various defects on the HRIPCB.

	YOLO11n			FMW-YOLO		
Category	P/%	R/%	mAP/%	P/%	R/%	mAP/%
All	94.9	87.6	92.8	95.1	90.5	94.9
Missing_hole	98.6	99.8	98.9	98.7	99.9	99.4
Mouse_bite	92.3	80.7	85.7	84.5	85.5	90.6
Open_circuit	94.7	85.1	95.4	98.5	90.6	97.8
Short	93.3	95.7	96.5	95.8	98.1	98.3
Spur	95.4	86.2	89.9	96.7	84.0	92.6
Spurious_copper	95.2	78.1	90.1	96.4	84.5	90.8

**Table 3:** Inference performance comparison.

Model	Preprocess (MS)	Inference (MS)	Postprocess (MS)	FPS	Size (MB)
YOLO11n	0.1	1.0	0.9	500.0	5.5
FMW-YOLO	0.1	0.9	0.6	625.0	4.5

**Table 4:** Backbone comparison.

Network Name	P/%	R/%	mAP50/%	mAP50-95/%	Params/M	GFLOPs
YOLO11n	94.9	87.6	92.8	48.5	2.58	6.3
VanillaNet	90.6	85.9	89.4	44.2	23.69	95.2
Unireplknet	84.1	76.0	80.8	38.6	5.80	14.1
Swin Transformer	89.8	84.3	87.7	43.7	29.72	77.6
STaRNet	90.1	78.7	86.2	42.3	1.94	5.0
HGNetV2	92.8	84.5	90.7	46.9	2.14	5.7
FCT-LFNet (ours)	96.4	89.9	93.7	48.9	2.57	6.6

**Table 5:** Neck comparison.

Network Name	P/%	R/%	mAP50/%	mAP50-95/%	Params/M	GFLOPs
YOLO11n	94.9	87.6	92.8	48.5	2.58	6.3
GDSAFusion	94.7	87.3	92.1	46.7	3.79	12.6
SDI	95.3	86.7	92.5	48.6	2.63	6.7
Gold-YOLO	94.4	88.5	92.4	47.3	5.90	9.2
GLSA	95.5	89.0	93.3	48.4	3.74	8.6
CSFCN	90.9	85.5	88.7	44.8	2.97	7.2
MSCAE (ours)	94.3	89.3	93.6	49.7	1.99	5.9

**Table 6:** IoU comparison.

IoU	P/%	R/%	mAP50/%	mAP50-95/%
Complete-IoU Loss	93.5	90.3	94.2	49.5
Scylla-IoU Loss	95.0	89.2	93.7	49.2
Distance-IoU Loss	94.6	89.7	93.8	49.0
Shape-IoU Loss	94.9	89.8	94.3	50.0
Efficient-IoU Loss	95.3	90.2	94.5	49.4
Wise-IoU Loss	95.1	90.5	94.9	50.0

**Table 7:** Data comparison for each model on the HRIPCB.

Model	P/%	R/%	mAP50/%	mAP50-95/%	Params/M	GFLOPs
Fast R-CNN [54]	93.3	81.1	88.3	41.6	23.59	136.8
SSD [55]	79.4	71.8	76.3	39.7	24.28	—
RT-DETR [2]	96.5	91.0	94.5	49.6	19.88	57.0
EfficientDet [56]	81.8	79.2	82.0	—	10.3	18.4
YOLOv3 [57]	—	—	92.5	44.2	61.5	18.9
YOLOv5 [58]	88.1	93.6	92.5	—	7.1	15.2
YOLOv7 [59]	88.3	84.6	87.3	—	71.3	103.2
YOLOv8s [60]	95.1	89.0	92.3	—	9.83	28.4
YOLO11n	94.9	87.6	92.8	48.5	2.58	6.3
FMW-YOLO	95.1	90.5	94.9	50.0	1.98	6.2

**Table 8:** Ablation study results.

Combination	P/%	R/%	mAP50/%	mAP50-95/%	Params/M	GFLOPs
YOLO11n	94.9	87.6	92.8	48.5	2.58	6.3
YOLO11n+F	94.7	89.9	93.7	48.9	2.57	6.6
YOLO11n+M	94.3	89.3	93.6	49.7	1.99	5.9
YOLO11n+F+M	93.5	90.3	94.2	49.5	1.98	6.2
YOLO11n+F+M+W	95.1	90.5	94.9	50.0	1.98	6.2

**Table 9:** Inference speed comparison of the DRRM and its variants.

Model	Preprocess (MS)	Inference (MS)	Postprocess (MS)	FPS
YOLO11n	0.1	1.0	0.9	500.00
+DWR	0.1	4.8	0.6	181.82
+DRB	0.5	1.1	0.7	434.78
+DRRM	0.1	0.4	0.9	714.92

**Table 10:** Comparison of YOLO11n and FMW-YOLO for various defects on the DeepPCB.

	YOLO11n			FMW-YOLO		
Category	P/%	R/%	mAP/%	P/%	R/%	mAP/%
All	98.3	95.6	98.6	98.4	95.9	98.9
Missing_hole	99.3	97.4	99.4	99.0	97.8	99.5
Mouse_bite	98.0	95.0	98.4	98.1	95.3	98.7
Open_circuit	97.8	97.2	99.1	97.6	97.9	99.5
Short	98.7	98.0	99.4	98.9	98.3	99.6
Spur	97.8	91.1	97.2	98.2	92.3	97.6
Spurious_copper	98.4	95.0	98.3	98.7	95.6	98.6

**Table 11:** Data comparison for each model on the DeepPCB.

Model	P/%	R/%	mAP50/%	mAP50-95/%	GFLOPs
Fast R-CNN [61]	97.24	96.91	97.41	73.74	201.3
SSD [31]	96.1	95.3	94.5	69.7	276.0
RT-DETR [48]	95.16	92.75	96.81	74.58	57.0
EfficientDet [62]	88.96	79.21	88.24	67.38	24.9
YOLOv3 [63]	92.45	92.68	95.86	66.78	154.6
YOLOv8s [64]	95.82	93.28	96.53	76.13	28.5
YOLOv10n [55]	94.9	93.9	98.1	76.9	---
YOLO11n	98.3	95.6	98.6	78.5	6.3
FMW-YOLO	98.4	95.9	98.9	78.9	6.2

**Table 12:** Performance stability under different random seeds on the DeepPCB.

Seed	YOLO11n	FMW-YOLO
0	98.6	98.9
42	98.6	99.0
188	98.5	98.8
1666	98.7	99.1
3407	98.5	98.8
Mean ± Std	98.58 ± 0.10	98.92 ± 0.12

## Data Availability

The data presented in this study are available on request from the corresponding author.

## Abbreviations

The following abbreviations are used in this manuscript:

PCB	Printed Circuit Board
AOI	Automated Optical Inspection
FPN	Feature Pyramid Network
SSD	Single Shot Detector
DFCAA	Dual-Frequency and Channel Attention Aggregation
LEGB	Lightweight Edge-Gaussian Block
FCT-LFNet	Frequency-Enhanced Channel-Transposed and Local Feature Network
MSFPNICA	Multi-Scale Feature Pyramid Network with Integrated Channel Attention
DRRM	Dilated Reparam Residual Module
MSCAE	Multi-Scale Context-Aware Enhancement
CA	Channel Attention
CT2d	ConvTranspose2d
CTA	Channel Transposed Attention
DFFN	Dual-Frequency Feed-Forward Network
DWConv	Depth-Wise Convolution
AN	Activation–Normalization
GAP	Global Average Pooling
SFF	Selective Feature Fusion
RR	Region Residualization
SR	Semantic Residualization
BN	Batch Normalization
DWR	Dilation-Wise Residual
DRB	Dilated Reparam Block
IoU	Intersection over Union
Mh	Missing_Hole
Mb	Mouse_Bite
Oc	Open_Circuit
Sh	Short
Sp	Spur
Sc	Spurious_Copper
FPS	Frames Per Second
R	Recall
P	Precision
AP	Average Precision
mAP50	Mean Average Precision
